# Provider-Recommended Strategies to Support Caregiver Needs After Inpatient Rehabilitation for Pediatric TBI

**DOI:** 10.3390/bs16071073

**Published:** 2026-07-01

**Authors:** Sophia Nichols, Nicole Viola, Angela Ciccia, Christine Koterba, Jennifer P. Lundine

**Affiliations:** 1 Department of Speech & Hearing Science, The Ohio State University, Columbus, OH 43210, USAlundine.4@osu.edu (J.P.L.); 2 Communication Sciences Program, Department of Psychological Sciences, Case Western Reserve University, Cleveland, OH 44106, USA; 3 Nationwide Children’s Hospital, Columbus, OH 43205, USA

**Keywords:** traumatic brain injury, pediatric, qualitative, rehabilitation, outcomes

## Abstract

Caregivers of children with traumatic brain injury (TBI) often report unmet healthcare needs following their child’s injury. The objective of this study was to survey medical and educational professionals in focus groups to respond to caregiver perceived gaps in care and service provision for children with TBI identified in a prior qualitative study. Subjects included 29 medical and educational providers. Reflexive thematic analysis of responses included inductive and deductive analysis and iterative consensus among the authors. Themes identified included (1) improving education and care in the hospital, (2) preparing families to go home, (3) improving behavioral health care, (4) navigating schools, (5) empowering caregivers with resources and (6) medical and system barriers. These solutions require implementation at the individual, hospital, and broader systemic levels. Overall, provider solutions illuminate a greater need to improve care coordination to navigate a child’s long-term medical needs and transition back to school, as well as the need to train more pediatric brain-injury-aware providers and improve policy and support systems.

## 1. Introduction

Traumatic brain injury (TBI) represents a leading cause of chronic disability in children and adolescents (Haarbauer-Krupa et al., 2018) with caregivers reporting unmet and unrecognized health care needs years after the initial injury (Dahl et al., 2024; Diener et al., 2022; Fuentes et al., 2018; Narad et al., 2019; Slomine et al., 2006). Further complicating matters, caregivers of children with TBIs lack informational, emotional, and structural support and have trouble accessing appropriate services, which contributes to unmet needs (Kirk et al., 2015; Lundine et al., 2023).

Care of children with TBI spans medical, educational, and community-based systems, but lacks centralized care coordination to help families navigate and access appropriate support (Davies et al., 2022; Lever et al., 2019; Lundine et al., 2023; Shook et al., 2022). Lack of coordination impacts long-term outcomes for children with TBI and makes it less likely they will receive outpatient rehabilitation services (Haarbauer-Krupa et al., 2017; Lundine et al., 2023). Without coordinated multidisciplinary care, caregivers report frustration as they are the sole advocate for their child to access appropriate services (Roscigno et al., 2015; Roscigno & Swanson, 2011).

Communication breakdowns between the medical system, school system, and caregivers for children with TBI are well documented and further contribute to reduced access to services and poorer long-term outcomes (Lundine et al., 2023). During initial interactions with medical providers, caregivers of children with TBI report feeling confused, misunderstood, and uninformed about their child’s TBI (Lundine et al., 2019; Roscigno & Swanson, 2011). Confusion persists when caregivers attempt to navigate the transition back to school. Families report that educators do not understand the realities of having a student with a TBI or of creating TBI specific educational plans (e.g., McCart et al., 2023). Families report that limited information is shared between hospital and schools and, therefore, schools often struggle to implement hospital recommendations (Todis et al., 2018).

### The Current Study

The literature is clear that partners in pediatric TBI are falling short in their efforts to integrate systems of care and improve post-injury outcomes. Taken together, there is an obvious need for evidence-based interventions that adopt a multidisciplinary perspective and prioritize collaborative problem solving. The current study represents the second phase of a participatory action research project that solicits input from key stakeholders in pediatric TBI to address perceived gaps in care. The goal of this phase was to gather qualitative recommendations from educators and healthcare professionals, focusing on practical solutions to meet caregiver-identified needs and improve services for children with TBI.

To better understand gaps in care, the first phase of the larger research project (Lundine et al., in press) used semi-structured interviews with 19 caregivers of children who were admitted to inpatient rehabilitation following a TBI. All youth experienced a TBI on average 5 years before the caregiver interview, so caregivers had time to reflect on their experiences in the hospital, the training they received, and how their life changed since their child’s injury. In the first phase of the study, researchers identified four major caregiver themes: (1) areas of lasting change in the child, (2) the overwhelming nature of the healthcare environment, (3) shifts in caregiver responsibility, and (4) long-term challenges for their child in school.

After analyzing caregiver interviews and themes, researchers designed phase two (the current study). Researchers translated each theme from the first phase into a corresponding, action-oriented “gap in care” for use in the second phase. This process involved a collaborative discussion to rephrase the descriptive themes as modifiable areas of healthcare delivery that providers could address during the focus groups. Pivoting from “themes” to “gaps in care” was not to reinterpret or alter the underlying findings, but to present the findings from phase 1 in a format that would encourage solution-oriented discussion and actionable recommendations during phase 2. Each gap in care directly reflected a caregiver-identified theme from phase 1: (1) improving education and care in the hospital (derived from theme 1: the overwhelming nature of the healthcare environment), (2) preparing families to return home (derived from theme 2: challenges related to shifting caregiver responsibilities), (3) improving mental health care (derived from theme 3: areas of lasting change in the child), and (4) navigating the school system (derived from theme 4: long-term challenges for their child in school).

## 2. Methods

### 2.1. Study Population

Researchers recruited focus group participants using purposive sampling to ensure that disciplines with specialized knowledge or experience in pediatric TBI were represented. Additionally, providers were purposefully sampled from the same midwestern pediatric hospital where caregivers were recruited to ensure they had knowledge of the same rehabilitation experience. To ensure a diverse representation of medical providers across disciplines, researchers sent targeted recruitment emails to the physical medicine and rehabilitation, neurosurgery, trauma, and rehabilitation teams. For educational stakeholders, the study team purposefully sent recruitment emails to two school psychologists from the local public-school district’s TBI Team and two public school teachers with experience on the inpatient rehabilitation unit at the same hospital. All participants met the following eligibility criteria: a licensed professional who has worked in pediatric TBI for at least one year and within the last five years; conversational speaker of English.

### 2.2. Research Description/Reflexivity Statement

The lead author of this work is a licensed speech–language pathologist and PhD candidate with 7 years of clinical and research experience. At the time of analysis, the second author was a PhD candidate and speech–language pathologist with 4+ years of direct clinical experience with individuals post-TBI. The remainder of the research team are researcher-clinicians, each with over 15 years of experience with pediatric TBI. The third and last authors (and focus group leaders) had worked (or currently work) clinically with all focus group participants and also work/worked clinically on the inpatient rehabilitation unit. Our team’s clinical background provided a meaningful framework for understanding the systemic and structural barriers that families and providers encounter. While this familiarity facilitated rapport with clinician participants and allowed for nuanced consideration of participant contributions, we remained attentive to the risk that familiarity might skew our interpretation of the focus group discussion.

None of the researchers had experienced pediatric TBI as a patient or caregiver, positioning us as outsiders to the lived experience of families navigating this system. We acknowledge that this outsider status may limit our ability to fully comprehend the emotional and logistical dimensions of care gaps as experienced by families. Our shared commitment to improving outcomes for children with TBI motivated this research. We recognize that this motivation carries the risk of confirmation bias. We addressed this potential for bias by including participants in both phases of the larger project who expressed satisfaction with current care pathways alongside those who recognized significant gaps.

### 2.3. Data Collection and Transcription

The third and last authors led focus groups with medical and educational providers over Zoom. Each focus group lasted approximately 60 min. After reviewing consent and privacy information, focus group leaders used PowerPoint and screensharing to show slides that reflected the four previously described gaps in care. Slides also included several caregiver quotes reflecting their thoughts in their own words.

The focus group leaders facilitated group discussion and allotted 10 min for participants to discuss each gap. Focus group leaders divided tasks such that one leader managed the slide show, and the other took fieldnotes, managed the time, and asked follow-up questions as appropriate. Once all gaps were discussed, the leader asked one last question for the final 10-min segment: “If resources weren’t an issue and you were in charge, what would be your ideal approach to delivering health care to children with TBI and their families?” Suggestions in response to this question were integrated into the gap in care or barrier that they addressed. Focus groups were recorded for later review and transcription. Following the fifth focus group, leaders agreed that no new ideas were being introduced, thus suggesting data saturation had been reached. The focus group guide is included as Supplementary Material.

All focus groups were transcribed by undergraduate research assistants. Original transcripts were verified by a second undergraduate research assistant who re-watched the video in its entirety, comparing it to the transcript and making edits when appropriate. For meaningful conflicts identified during the reliability checking, the last author (JL) re-watched the video and resolved questions.

### 2.4. Analysis

Researchers selected a constructivist paradigm to guide the current study. Constructivists understand meaning as co-constructed through interactions and shaped by the individuals’ contexts, making it well suited for focus group research (Appleton & King, 2002). Constructivism aligns with the current study’s aim to identify actionable solutions as it emphasizes shared meaning-making through interaction and the integration of multiple stakeholder perspectives within applied, real-world contexts. Within this paradigm, researchers employed a participatory action research methodology (Baum et al., 2006). Participatory action research is a collaborative approach that engages stakeholders as active partners in the research to generate results that are directly applicable to improving practice and promoting meaningful change.

**Figure 1:**
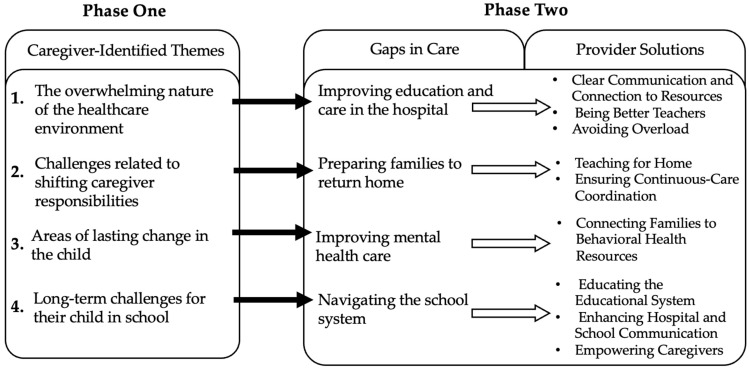
Integration of Phase 1 and Phase 2 Outcomes.

Researchers predominantly utilized a deductive approach to data analysis using the four caregiver-identified gaps in care as the initial coding framework. Within each gap, an inductive approach was also used to identify novel strategies and recommendations that emerged from participants’ discussions, ensuring that educators’ and healthcare professionals’ perspectives were represented. Inductive analysis followed the steps of reflexive thematic analysis (Braun & Clarke, 2019, 2022, 2023). After importing the transcripts into Nvivo 14 (Lumivero, 2023), a qualitative software for organizing materials, the first and second authors read the five transcripts in their entirety to familiarize themselves with the data. On their second reading of the transcripts, the first and second authors coded transcripts independently, generating a list of initial codes and combining these codes into solutions to the proposed gaps in care. This stage involved note-taking and reflexive memoing as researchers noted their own thinking processes to describe how codes were determined. Once the researchers each believed that their list of novel codes was exhausted, the first author examined both codebooks to determine areas of disagreement and agreement. While maintaining all earlier versions of codebooks, she first proposed a combined codebook that was then discussed with the first, second, and last authors until consensus on a final codebook was reached. This iterative process generated overarching solutions for each gap in care, alongside specific strategies for their implementation. To support the trustworthiness of the qualitative findings, select quotations for each strategy were re-read by all authors to affirm that the specific strategies and overarching solution accurately reflected what the providers had discussed in the focus groups. Figure 1 reflects this analytic process and the outcomes.

During data analysis, the research team observed that the fifth focus group contributed minimal novel strategies beyond what had been identified in earlier discussions. This suggested that the major solutions had been captured within the available sample and data saturation had been reached. The number of focus groups also fits within established guidelines expected for focus groups to meet data saturation (Hennink & Kaiser, 2022).

## 3. Results

### 3.1. Sample Characteristics

Table 1 displays the demographic and professional details for each of the 29 participants across the five focus groups. Focus groups ranged in size from four to eight people. Participants averaged 14.5 years of clinical, medical, or educational practice (with an average 13.25 years in pediatric TBI, specifically). Most participants (79%) were female, and while all participants had at least a bachelor’s degree, most had a master’s degree (31%) or higher terminal degree (48%; e.g., PhD, MD).

### 3.2. Provider-Proposed Solutions

In focus groups, leaders facilitated discussion based on four gaps in care and asked medical and educational providers to brainstorm actionable solutions. The gaps in care discussed in each focus group were: (1) improving education and care in the hospital, (2) preparing families to return home, (3) improving behavioral health care, and (4) navigating the school system. As researchers reviewed transcripts, consistent solutions were identified when there was agreement across disciplines and focus groups. Providers also identified barriers to implementing these solutions, which researchers felt merited inclusion as a separate theme: provider-perceived barriers. Exemplar quotes are included in Table 2.

### 3.3. Gap 1: Improving Education and Care in the Hospital

#### 3.3.1. Provide Clear Communication and Connection to Resources

Providers recognized that information moves rapidly in the hospital and comes from many different disciplines. They proposed three strategies to improve communication and better connect families to resources.

1. A point person for questions and resources: provide a consistent individual to advocate for families, coordinate information, and support care transitions.
2. Consistent communication: regularly schedule provider care conferences to provide a consistent message to families regarding prognosis and treatment planning.
3. Care team introductions: repeatedly introduce care team members and explain each provider’s role; provide visual references (e.g., pictures with provider names and roles on the patient’s wall).

#### 3.3.2. Be a Better Teacher

Providers were aware they could modify their teaching style and better equip families with the knowledge they need to successfully understand and navigate pediatric TBI while families are in the hospital.

1. Include the parent in their own learning: invite caregivers to participate in therapy sessions, empower caregivers to ask questions, and explain the intention behind therapeutic activities.
2. Employ specific teaching strategies: adjust to the caregivers’ preferred learning style, simplify messages into smaller pieces, constantly repeat information, and use teach-back methods to verify caregivers’ understanding.
3. Provide knowledge-tracking materials: use tools, such as care journals, to document strategies the caregivers have learned and education they have received so the team knows which remaining items need to be taught before discharge.

#### 3.3.3. Avoid Overload

Despite the perceived clinical value of providing education as early as possible, providers recognized that families vary in their readiness to receive information and generated strategies to avoid overwhelming families.

1. Assess family readiness: assess indicators of readiness including initial caregiver behavior with the provider (e.g., are caregivers engaging with the provider or receptive to the interaction?), the caregiver’s response to any physical materials, and the tenuousness of their child’s prognosis.
2. Wait to educate, when possible: allow caregivers to decide when they would like to receive education, with an optimal window during the middle of a hospital stay. However, providers noted that if education is necessary early in the hospitalization, messages should be simplified to emphasize key points. Providers should also give one piece of information at a time.

### 3.4. Gap 2: Preparing Families to Go Home

#### 3.4.1. Teach for Home

Medical and educational providers recognized that improving pre-discharge education was an essential part of helping caregivers feel better equipped to transition home.

1. Educate caregivers on the realities of home life: compare home and hospital life and repeatedly emphasize that caregivers will have to do what the hospital staff do in addition to their preexisting responsibilities as caregivers at home (e.g., employment, caring for other children).
2. Plan for home realities: schedule care conferences aimed at anticipating family barriers and daily care needs to develop concrete discharge plans and recommendations.
3. Provide sufficient hands-on experience prior to discharge: give multiple opportunities for families to independently practice caregiving in a real-world setting, with as little support from the medical team as possible. Providers viewed this as a form of “home practice” after which families could work with hospital staff to resolve outstanding areas of need before their final discharge.

#### 3.4.2. Ensure Continuous Care Coordination

Providers recognized that outpatient care for pediatric TBI is sparse compared to the specialized intensive care that families receive in inpatient settings or that children with other complex medical conditions receive. Providers acknowledged it is essential to better connect families with the resources they need to be successful long-term.

1. Facilitate TBI care for TBI patients: educate outpatient providers on best practices for treatment of children post-TBI; establish outpatient clinics dedicated to children with brain injury; create specialized day treatment programs that act as a step-down from inpatient care.
2. Provide dedicated follow-up services: create a position for a personalized care coordinator (also described as a case worker) who could help navigate the transition home by serving as the intermediary between families and the medical system, checking in on families, coordinating appointments and transportation, and connecting families with community resources.

### 3.5. Gap 3: Improving Behavioral Health Care

#### Connect Families to Behavioral Health Resources

Providers discussed how more timely, accessible, and specialized behavioral health care could address caregivers’ lack of preparedness for their child’s post-TBI behavioral health needs.

1. Make behavioral health a top priority: start discussing behavioral health as early as possible with families and emphasize that their child and family may benefit from support adjusting to post-injury life; gauge family openness to behavioral health care.
2. Coordinate behavioral health care from the hospital: establish follow-up behavioral health care before discharge, provide local behavioral health resources, and/or reconnect with families after discharge to continue to offer services as new needs might arise once they are home.
3. Find a community of care: connect families to community and social supports including respite care, mentorship, and peer groups. If a comprehensive outpatient clinic is established, it could include behavioral health providers who are specialists in TBI to provide ongoing support following discharge.

### 3.6. Gap 4: Navigating the Schools

#### 3.6.1. Educate the Educational System

Given parent frustrations with the school’s lack of TBI awareness, providers strategized ways to better educate school personnel on the realities of having a student with a TBI in their classrooms.

1. Raise awareness through trainings and resources: educate schools through presentations by clinicians or families, video modules, professional development opportunities and advocate for adding pediatric TBI to the curriculum for future educators. Helpful resources for educators included clinical notes on maximizing student performance and comprehensive lists of potential accommodations.
2. Think long-term: regularly assess children with a history of TBI as their needs may change over time and be aware of how recovery following pediatric TBI is unpredictable. Testing may be most important at times of transition from elementary to middle school and middle to high school.

#### 3.6.2. Enhance Hospital and School Communication

Providers shared caregiver frustrations on the lack of communication between the school and medical system and identified their role in enhancing communication.

1. Share medical information with schools: establish communication with schools as early as possible with medical releases of information and schedule regular school reintegration meetings including families, the medical team, and relevant school personnel. During meetings, educators and hospital staff could discuss the child’s needs, related resources, and recommended accommodations. Designate a hospital contact person to help answer questions from the school, during and after the hospitalization.
2. Encourage school involvement before discharge: ask for a school representative to follow the child in the hospital and provide more regular updates to the school as the child progresses throughout their admission.

#### 3.6.3. Empower Caregivers with Resources

In addition to establishing contact between the medical team and the school, providers highlighted the need to help caregivers navigate the school system after their child’s TBI.

1. Empower caregivers: connect them with advocates or mentors and inform them of their educational rights; advocate for state funded care coordination to ensure caregivers and children are connected to appropriate educational resources.

### 3.7. Provider Perceived Barriers to Care

In addition to generating solutions to address caregiver-perceived gaps in care, one deductive theme was also identified through qualitative analysis of focus group transcripts: barriers to care. Throughout each group, providers consistently highlighted barriers at both the patient/family level and within the medical system that make it hard to address gaps in care.

#### 3.7.1. Patient and Family Barriers

1. TBI is an invisible injury. Providers expressed that caregivers and school personnel may interpret the physical recovery as representing a full recovery and thus the end of the child’s therapeutic needs. Likewise, for children with lasting physical disabilities, caregivers and school personnel may prioritize these visible needs over “unseen” disabilities such as cognitive or behavioral health challenges. A focus on physical recovery status may result in reduced efforts to establish or seek services for lasting cognitive and behavioral needs. Similarly, providers discussed how hard it is to explain that TBI symptoms might change over time and across development, which then might delay appropriate care in the future.
2. Families face unique stress and burdens. Providers highlighted how caregivers experience stress in managing their child’s medical care and can feel overwhelmed with new information and fears for their child. Providers acknowledged that financial stressors (e.g., increased pressure to maintain employment while caring for a child with medical needs, new medical expenses, transportation challenges) may prevent families from being available for both inpatient and outpatient therapy sessions, creating barriers to not only education but overall medical care. Unique stressors may lead to diminished follow-up care or make caregivers feel cognitively overwhelmed during their child’s admission and appointments. Stressors may also contribute to caregivers being less able to take in education, for example, while their child is hospitalized.

#### 3.7.2. Medical and System Barriers

1. Provider schedules limit accessibility and flexibility. Demanding schedules make it difficult for providers to devote as much time as they would like to patient care and additional meetings, such as care conferences or school reintegration meetings. Busyness also likely limits the number of providers who are willing and able to see children with TBI, leading to long waitlists for outpatient care.
2. Insurance limits access to care. Insurance coverage may dictate what services and providers are approved/denied which could impact access to care and lengthen already long wait times. Providers often feel pressure from insurance companies to discharge as soon as possible, even at times when families or the medical team may not feel the child is ready to return home yet.
3. COVID-19 changed healthcare. During the pandemic, providers were limited in their ability to perform pre-discharge home or school visits. Providers also discussed that they believed the COVID-19 pandemic led to increased educator stress and burden, which in turn may have affected their ability to meet the needs of students with TBIs.

## 4. Discussion

In this study, researchers analyzed focus group discussions with medical and educational stakeholders to identify solutions to caregiver-perceived gaps in care for children with TBI. Medical and educational providers identified the following categories of actionable solutions: (1) provide clear communication and connection to resources, (2) be better teachers, (3) avoid overload, (4) teach for home, (5) ensure continuous care coordination, (6) connect families to behavioral health resources, (7) educate the educational system, (8) enhance hospital and school communication, and (9) empower caregivers. These findings are consistent with previous studies and offer new insights for future participatory research focusing on the need for better communication and teaching, intentional preparation for home and school transitions, and mental health support for children with TBI and their families.

### 4.1. Improving Education and Care in the Hospital

Provider recommendations to improve communication, resource access, and hospital-based education reinforce a consistent finding across the literature: families benefit from clear, coordinated, and responsive information delivery (Roscigno et al., 2013; Sisk et al., 2021). Findings from this study, combined with past research, suggest that gaps in communication and education are not due to a lack of known strategies, but rather to challenges in implementation within time- and resource-constrained healthcare settings. Importantly, improving these aspects of care may have downstream effects beyond the inpatient experience, including better engagement with follow-up care (Lever et al., 2019). From an implementation perspective, providers in this study offer suggestions that highlight the need for system-level approaches that support providers in delivering consistent, high-quality education, such as structured communication protocols, dedicated roles for care coordination, and integration of reliable educational resources into routine care workflows (Palusak et al., 2022; Shook et al., 2022). Future research should focus on testing scalable interventions that embed these strategies into clinical practice, as well as evaluating broader outcomes, including care coordination and following families past the point of hospital discharge.

### 4.2. Preparing Families to Return Home

Provider recommendations in this study highlight discharge planning as a critical yet underdeveloped area of care. The existing evidence, however, suggests that more comprehensive and structured discharge support improves caregiver understanding and increases follow-up engagement (Carroll et al., 2024; Glick et al., 2017; Keenan et al., 2020), indicating that current gaps are less about lack of effective strategies and more about inconsistent implementation. Despite the understandable prioritization of acute symptom management during hospitalization, inadequate preparation for the transition home may undermine the benefits of inpatient care. Findings from this study underscore the importance of embedding standardized, family-centered discharge practices into routine care, such as clear written instructions, proactive follow-up supports, and coordination across medical and educational systems. From an implementation standpoint, integrating these practices into existing workflows will be essential to ensure feasibility in busy clinical settings and effectiveness for families once they return to “real life.” Future research should focus on developing and testing scalable discharge interventions that strengthen care transitions and evaluate their impact on long-term outcomes and healthcare utilization and access for youth with TBI.

### 4.3. Meeting Behavioral Health Needs

Taken together with prior research, provider recommendations in this study reinforce that unmet behavioral health needs remain a persistent and consequential gap in pediatric TBI care for both children and their caregivers (e.g., Fisher et al., 2021). The consistency of provider recommendations with the existing literature suggests that effective strategies such as earlier behavioral health intervention (Narad et al., 2019), integration into multidisciplinary care (Miley et al., 2022), and connection to peer and community supports (Ergh et al., 2002; Hanks et al., 2012; Hibbard et al., 2002; Sartore et al., 2021; Witten et al., 2025) are well established but not routinely implemented. As a result, behavioral health continues to be insufficiently prioritized despite its clear influence on long-term child outcomes and overall family functioning (Anderson et al., 2009; Micklewright et al., 2012; Moscato et al., 2021; Raj et al., 2013; Taylor et al., 2002). Effectively addressing the behavioral health needs of youth with TBI and their caregivers will require system-level changes that improve access to and integration of behavioral health services, including enhanced provider training, expanded coverage mechanisms (e.g., Medicaid waivers, increased insurance coverage for behavioral health services), and incorporation of behavioral health into outpatient care. Future research should focus on developing and testing scalable approaches that embed these supports across the care continuum, particularly during the transition from hospital to home, to better support sustained recovery and family well-being.

### 4.4. Returning to School

New placements into special education rarely occur beyond the first-year after a TBI (Taylor et al., 2003), thus making the transition from the hospital to school a critical inflection point for long-term outcomes after pediatric TBI. Despite this, the responsibility for coordinating this transition often falls disproportionately on caregivers (Haarbauer-Krupa et al., 2017; Lundine et al., in press). The consistency between this study and the prior literature suggests that effective components of successful school reintegration, such as clear communication across systems, defined points of contact, and ongoing monitoring, are well established but not systematically implemented (Davies et al., 2022; Haarbauer-Krupa et al., 2023; Palusak et al., 2022; Unruh et al., 2023). Persistent gaps appear to reflect fragmentation across healthcare and educational systems rather than a lack of known solutions. Improving return-to-school outcomes will require coordinated, cross-system approaches that formalize communication between medical teams, schools, and families, while clarifying roles and expectations across settings (Davies et al., 2022). Establishing dedicated points of contact and increasing TBI-specific training for school personnel may help reduce reliance on caregivers as intermediaries. Future research should focus on developing and testing scalable models of coordinated return-to-learn systems that align stakeholders, improve access to school-based supports, and ultimately enhance long-term academic and functional outcomes for children with TBI.

### 4.5. All Roads Lead to Care Coordination

While care coordination itself was not evaluated as a specific intervention in this study, it emerged as a central recommended strategy across focus group discussions. McDonald et al. (2007) define care coordination as “the deliberate organization of patient care activities between two or more participants (including the patient) involved in a care to facilitate the appropriate delivery of health care” (p. 41). Care coordination in various forms was discussed as a vital tool at and between all levels of care. Recommendations to facilitate communication between agencies, provide personnel to navigate transitions, supply educational resources, and promote easier access to specialized providers are all examples of care coordination. These solutions would improve the availability and quality of post-TBI health care thus greatly reducing the burden on the caregiver. However, this paper is one of many calling for greater levels of care coordination in improving the long-term outcomes of children with TBI (e.g., Davies et al., 2022; Haarbauer-Krupa et al., 2017; Lundine et al., 2023; Palusak et al., 2022; Shook et al., 2022). Care coordination has the potential to minimize caregiver perceived gaps in care and reduce the strain on medical and educational providers, ultimately improving access to care and securing better long-term outcomes for children with TBI.

### 4.6. Overcoming Barriers to Proposed Solutions

The literature has consistently reported similar gaps in care for decades. This begs further inquiry into the barriers preventing meaningful improvement. While the barriers are consistent with those previously identified in the literature, this study is unique in that the barriers come directly from the end-user charged with problem solving gaps in service delivery. Another study identified the same family stressors (e.g., employment stress, time demands and limited resources) and challenges with insurance coverage as primary barriers to behavioral health care following pediatric TBI (Miley et al., 2022). In addition to these patient and medical-system factors, the invisibility of the TBI itself continues to undermine long-term care. A survey of caregivers of children with TBI found that demonstrating “no-need” was the most common barrier to pursuing follow-up care (Lever et al., 2019). Given the limited ability of caregivers to recognize late emerging cognitive or behavioral symptoms related to the TBI (Greenspan & MacKenzie, 2000; Slomine et al., 2006), these findings may speak to the “invisibility” of pediatric TBI, rather than the lack of “need.” The consequences of hidden needs in pediatric TBI become more apparent when we compare physical rehabilitation services to behavioral health and/or cognitive services, where the latter services are less likely to be reimbursed, less available, and less frequently accessed (Hoffmann et al., 2022; The Kennedy Forum, 2026). These persistent barriers are likely undermining health care utilization and access for children with TBI and their families, despite these being areas of greatest need (Fuentes et al., 2018). Future intervention research must consider these barriers in both study design and outcome evaluation. Incorporating barriers identified by medical and educational providers, the stakeholders responsible for implementing interventions, will be critical for driving system-level change.

### 4.7. Limitations

Barriers to care and unmet health needs are the result of an intricate web of factors, not all of which are directly within the scope of medical and educational providers. For example, compromised family functioning, low socioeconomic status, having Medicaid coverage, and being of a minority race are also associated with experiencing unmet health care needs post-pediatric TBI (Fisher et al., 2021; Fuentes et al., 2018; Narad et al., 2019; Slomine et al., 2006). These larger societal factors may not have been adequately considered in this study by educational and hospital staff and deserve further examination as contributing factors to these complex challenges moving forward.

Additionally, all caregivers who pointed out gaps in care and helped guide the focus group discussions had children who received inpatient rehabilitation. In addition to receiving inpatient care, their children were predominantly male and the majority had severe TBIs. Their perspectives, therefore, may not be generalizable to all caregivers of children with TBI. Learning from diverse perspectives is key as researchers and stakeholders work to implement meaningful and lasting change. The providers in the current study are from the same large midwestern medical center from which caregivers in the first phase of the study sought treatment. The single center design of this project may narrow the breadth of perspectives as both providers and caregivers shared an institutional context and were therefore reflecting on experiences within the same healthcare setting. This may also limit the transferability of our findings to healthcare settings outside of where the research was conducted. Some of this homogeneity may be mitigated by the hospital’s location in the central part of the state, and the recruitment of families from rural, suburban, and urban areas with some socioeconomic diversity. Nevertheless, the inclusion of multiple sites and a more geographically diverse sample would likely enhance the breadth of perspectives and strengthen the transferability of findings to other healthcare contexts, including international settings. Additionally, future work should incorporate policymakers, administrators, and insurers who also may influence the practicality and implementation of any proposed solutions to care provision.

## 5. Conclusions and Future Directions

The current study offers practical recommendations by medical and educational end-users to improve quality of and access to care from medical and educational providers who work within the systems that support children with TBI. Specifically, this study relied on caregiver perspectives to illuminate the persistent challenges experienced by families. Larger systemic solutions will require overcoming significant barriers such as funding and policy and will require greater resources and time. The importance of care coordination emerged as holistic solution encompassing many, if not all, of the provider-recommended solutions. Future work should prioritize securing funding to pilot this position and evaluate whether families who receive this support report fewer unmet needs and greater satisfaction compared to families who do not receive these services. Building on specific strategies recommended in this study, future research should develop and evaluate scalable approaches that embed improved educational training for caregivers, discharge practices, behavioral health supports, and medical-school transitions based on the feedback from end users and other key stakeholders.

## Figures and Tables

**Table 1:** Participant Education and Professional Experience.

Focus Group Participants	Total yrs. Practicing	pTBI Experience	Sex	Focus Group	Age	Highest Level of Education
Discharge Planner	19	13	Female	1	45	Bachelor’s Degree
Inpatient Nurse 1	22	22	Female	1	45	Bachelor’s Degree
Physical Therapist 1	37	35	Female	1	59	Bachelor’s Degree
Neuropsychologist 1	6	1.5	Female	1	33	Doctoral Degree (PhD)
Social Worker	7	5	Female	1	40	Master’s Degree
Occupational Therapist 1	35	35	Female	2	58	Master’s Degree
Speech Language Pathologist 1	18.5	18	Female	2	42	Master’s Degree
* Surgeon	5.5	7.5	Male	2	41	Professional Degree (MD)
Physical Therapist 2	5	5	Female	2	31	Professional Degree (DPT)
* Neurosurgeon	5	10	Male	2	44	Professional Degree (MD, PhD)
Child Life Specialist 2	9	9	Female	3	31	Bachelor’s Degree
Recreation Therapist	16	15	Female	3	39	Bachelor’s Degree
Teacher 1	38	33	Female	3	61	Master’s Degree
Speech Language Pathologist 2	16	15	Female	3	41	Master’s Degree
Physiatrist 1	6	12	Male	3	39	Professional Degree (MD)
School Psychologist 1	25	15	Female	3	49	Professional Degree (Ed.S.)
Occupational Therapist 2 *	5.5	7	Female	3	30	Professional Degree (OTD)
Psychologist 1 *	5	7	Female	4	35	Doctoral Degree (PhD)
Teacher 2	15	7	Female	4	39	Master’s Degree
Inpatient Nurse 2 *	13.75	14.75	Female	4	36	Master’s Degree
Nurse Practitioner *	24	25	Male	4	45	Master’s Degree
Outpatient Nurse 1	36	20	Female	4	56	Master’s Degree
Physiatrist 2 *	6	10	Male	4	39	Professional Degree (MD)
Physiatrist 3	6	5	Female	4	36	Professional Degree (MD)
Physiatrist 4	11	11	Male	4	43	Professional Degree (MD)
Outpatient Nurse 2	10	10	Female	5	61	Bachelor’s Degree
Psychologist 2 *	0.5	2.5	Female	5	30	Doctoral Degree (PhD)
Psychologist 3 *	11	12	Female	5	41	Doctoral Degree (PhD)
School Psychologist 2	8	2	Female	5	42	Doctoral Degree (PhD)

Note. pTBI Experience = years of experience in pediatric Traumatic Brain Injury (pTBI). * Indicates when years of experience with pTBI is higher than total years practicing, due to either previous lived, educational or other professional experiences.

**Table 2:** Solutions, Recommendations, and Associated Exemplar Quotes from Focus Groups.

Solution	Specific Recommendation	Exemplar Quote (Speaker)
Improving education and care in the hospital		
Clear Communication and Connection to Resources	Having a point person for questions and resources	“I’m going to be your resource, I may not have all the answers for you, but I can get them and I can find them” (Nurse Practitioner)
	Ensuring consistent communication	“Everyone from medical students all the way up to intern’s, resident’s, fellow’s, and attending’s that can give messages” (Surgeon)
	Introducing the care team	“I always introduce myself, … the service I’m with, even if I’ve seen this parent and the child 12 times” (Physiatrist 3)
Being Better Teachers	Including the parent in their own learning	“I would like you to be part of therapies and actually do things with me” (Inpatient Nurse 2)
	Employing specific teaching strategies	“Not just like giving the education but making sure that they’re receiving it” (Occupational Therapist 2)
	Providing knowledge-tracking materials	“Having something that I can go back in and check off” (School psychologist 1)
Avoiding Overload	Assessing family readiness	“you have to kind of gauge where the family is at, do they wanna ton of information is that too much?” (Speech Language Pathologist 2)
	Waiting to educate	“Sometimes it’s like literally them being like I can’t have this conversation right now” (Psychologist 3)
Preparing Families to Go Home		
Teaching for Home	Educating caregivers on the realities of home life	“Just let them know that things are going to look a lot different once they get outside these four walls of the hospital” (Physiatrist 3)
	Planning for home realities	“Our discharge planner, constantly asking what are the barriers, what are some strategies where we can provide more assistance” (Occupational Therapist 1)
	Providing sufficient hands-on experience prior to discharge	“Simulate real world experiences as much as possible” (Occupational Therapist 2)
Ensuring Continuous-Care Coordination	Identifying TBI care for TBI patients	“Getting better trained therapists that are really dedicated to the TBI population on our outpatient team” (Physical Therapist 1)
	Following the family	“I think that’s the disconnect too is like they go through these transitions of care um and there’s not like a single person that’s helping them” (Physical Therapist 2)
Improving Behavioral health Care		
Connecting Families to Behavioral Health Resources	Setting the priority	“It could just be a mandated or not mandated but a suggested recommended normal part of any treatment” (School Psychologist 2)
	Coordinating behavioral health care from the hospital	“it is part of the treatment plan upon discharge and even appointments set up before they leave” (Outpatient Nurse 2)
	Finding a community of care	“You have … support groups natural happens [at a] comprehensive clinic and I know I’m kind of beating a dead horse but it just works really well” (Neurosurgeon)
Navigating the Schools		
Educating the Educational System	Providing trainings, resources and raising awareness.	“meeting with the school therapist or providing education to the teachers and I know we do a school meeting to kind of hand off, but then it’s like okay this teacher is actually ready to work with this kid” (Speech Language Pathologist 2)
	Thinking long term	“Every couple years updating the neuro psych testing … if nothing else, just with a new group of teachers … maybe even a new building or new school if they’ve moved just to kind of reset hey, this is what you can do, this is how you can help the individual” (Physiatrist 4)
Enhancing Hospital and School Communication	Sharing information	“I think from a ground game standpoint communication with just a guidance counselor at each of these schools… I mean if they had a pipeline to our clinics you know we could answer some questions about you know is this appropriate is this not.” (Neurosurgeon)
	Encouraging involvement before discharge	“I update them throughout the student’s stay and then they are part of the discharge meeting if they can be and then parents know that they have a contact person who can go to the IEP meetings with them” (Teacher 1)
Empowering Caregivers	Empowering Caregivers	“I think encouraging the families to reach out for help when they’re not getting what they need from their school and then, would allow outreach maybe to that school regarding that individual situation” (Psychologist 2)

## Data Availability

The data that support the findings of this study are available from the corresponding author upon reasonable request.

## Supplementary Materials

The following supporting information can be downloaded at: https://www.mdpi.com/article/10.3390/bs16071073/s1. Focus Group Guide.

## Abbreviations

The following abbreviation is used in this manuscript:

TBI	Traumatic Brain Injury
