# Supplementary material for: Provider-Recommended Strategies to Support Caregiver Needs After Inpatient Rehabilitation for Pediatric TBI
Source: Behav Sci (Basel). 2026 Jul 1;16(7):1073. doi: 10.3390/bs16071073 (PMC13405689; doi:10.3390/bs16071073)
Supplement: Supplementary file 1 [file behavsci-16-01073-s001.zip › behavsci-4350601-supplementary.pdf]

<u>Identification of Critical Research and Intervention Needs in Pediatric Traumatic Brain Injury: Stakeholder Perceptions</u>

GUIDE for Focus Group with MEDICAL PROFESSIONALS

OVERVIEW OF INTERVIEW TOPICS

In this interview, I will ask you a series of open-ended questions to get your perspectives about different topics. As an overview, these topics are:

- Section 1: Introductions
- Section 2: Thoughts about hospital-related experiences
- Section 3: Thoughts about transitioning to home
- Section 4: Thoughts about counseling, mental health needs and physical/emotional changes in the child
- Section 5: Thoughts about school/educational experiences

Interviewer#1: Before we get started, please go up to the 3 dots on your picture, and edit your name to the way you would like to be addressed during the focus group. If it were me, I could put "Interviewer#1, since most of you know me as Interviewer#1 – but I could put Dr. Lundine as well."

We are going to record this meeting so that we can transcribe everyone's comments after the focus group is completed. ** I sent you the consent information, and it's here on the screen if you need to review it. I'm going to go through it very quickly before we begin.

** hit highlights**

Please go into the chat and type "yes" if you consent to participate in the study. If you choose not to consent, we appreciate your time, and you may leave the meeting now. I'm going to begin recording now.

Begin recording.

It is very important to respect the confidentiality of everyone in this group. Out of respect to others, please do not share the names of anyone in this group or repeat what we discuss outside of this focus group.

INTRODUCTION TO THE FOCUS GROUP

Interviewer#2: Section 1: in the interest of time, please put your occupation and which units you primarily work in in the hospital (5) also – when we get going with our discussion, please keep yourself muted until you want to share and feel free to jump in when you have something to add

Interviewer#1:

- Share purpose of study, short and long-term goals →
  - PowerPoint slide: Aim 1 and Aim 2 of study. *Our hope is that you can use your experience, combined with what we learned from caregivers, to help us determine next steps forward as we try to improve the long-term care for children and adolescents with TBI*
- We recently interviewed 20 caregivers of children who sustained a TBI and received medical treatment, including inpatient rehabilitation at NCH. We have analyzed transcripts from 16 of those interviews, and basic demographic and injury-related information is shown here. All of these children were admitted to our inpatient rehabilitation program. They range in age at the time of injury and current age. Also, we have a range of time since injury, so many of these families have lived with TBI for several years.
- From these interviews, we identified themes related to the following areas:
  - Hospital-related experiences
  - Transitioning to home
  - Counseling, mental health needs
  - School/educational experiences
- Our goal today is to gather ideas from you about how we might improve the continuum of care for children with TBI while hospitalized – and once they leave NCH.

Let's begin by talking about the experiences family shared about their time here in the hospital.

Interviewer#2: Section 2: Thoughts about hospital-related experiences (10 min)

- Share a few examples of quotes from these areas – here are some caregiver quotes related to their experiences in the hospital.
- Tell us your thoughts related to the education we provide to families while they are in the hospital – both immediately after injury (ICU) and longer-term as they are on the rehabilitation unit.
- Based on what we are hearing from parents, are there areas you feel could be improved? And if so, any ideas on how we might do that?

  - *What adjustments could you imagine in our education-provision, perhaps considering the timing and intensity of education provided? Or our assessment of how that information is received by families?*
  - *What barriers exist to meeting these needs and what are your ideas around how we might get around these barriers?*

Interviewer#1: Section 3: Thoughts about transitioning to home (10 min)

- Share a few examples of quotes from these areas

- Some caregivers shared that they experienced difficulties related to the transition from hospital to home or finding resources in their own community. As a team, we view discharge as an ongoing process, but it doesn’t seem like all families are experiencing it in the same way.
- Are there ways we could re-imagine the discharge process to make families feel more prepared to go home?

    - *How do we assess the challenges/barriers that families might face when they go home? What do you see as possible opportunities here?*
    - *Based on the quotes that we shared, what other ideas do you have surrounding this topic?*

Interviewer#2: Section 4: Thoughts about counseling, mental health needs and physical/ emotional changes in the child (10 min)

- Share a few examples of quotes from these areas
- Families are telling us that they are experiencing a need for additional mental health support – both for parents and also the survivor of TBI.
- How could we better prepare families to address longer term challenges their child might experience?

    - *Tell us your thoughts on how we could better address these concerns.*
    - *What barriers exist to meeting these needs and what are your ideas around how we might get around these barriers?*

Interviewer#1: Section 5: Thoughts about preparing school & educational experiences (10 min)

- Share a few examples of quotes from these areas.
- Families continue to talk about the challenges faced in receiving appropriate support from school personnel.
- What potential directions can you imagine to help us integrate the medical-school partnership to address these longer term needs?

    - *Tell us your thoughts on how we could better address these concerns.*
    - *What barriers exist to meeting these needs and what are your ideas around how we might get around these barriers?*

Interviewer#2: WRAP-UP (10 min) – could call on people if there’s no movement

- If resources weren’t an issue and you were in charge, what would be your ideal approach to delivering health care to children with TBI and their families?
    - *Is there anything you thought we would be discussing that we haven’t touched on?*

THANK YOU so much for your time and participation. Your comments were extremely helpful. We will be mailing out gift cards to thank you for your participation.
